# Maintenance therapy with once-monthly administration of long-acting injectable risperidone in patients with schizophrenia or schizoaffective disorder: a pilot study of an extended dosing interval

**DOI:** 10.1186/1744-859X-6-3

**Published:** 2007-01-29

**Authors:** Georges M Gharabawi, Natalie C Gearhart, Robert A Lasser, Ramy A Mahmoud, Young Zhu, Erik Mannaert, Ineke Naessens, Cynthia A Bossie, Mary Kujawa, George M Simpson

**Affiliations:** 1 Roche Pharmaceuticals, Nutley, NJ, USA; 2 Ortho-McNeil Janssen Scientific Affairs, LLC, Titusville, NJ, USA; 3 Comprehensive Neuroscience, Pennington, NJ, USA; 4 Medical Affairs, Janssen Pharmaceutica, Inc., Titusville, NJ, USA; 5 Johnson & Johnson Pharmaceutical Research & Development, Beerse, Belgium; 6 Department of Psychiatry, Keck School of Medicine of the University of Southern California, Los Angeles, CA, USA

## Abstract

**Background:**

Several clinical studies have established the efficacy, safety, and tolerability of long-acting risperidone administered once every 2 weeks in patients with schizophrenia or schizoaffective disorder. This report evaluates preliminary efficacy, safety, tolerability, and pharmacokinetic data for a novel (once-monthly) administration of long-acting injectable risperidone 50 mg in patients with schizophrenia or schizoaffective disorder.

**Methods:**

Clinically stable patients participated in a 1-year, open-label, single-arm, multicenter pilot study. During the 4-week lead-in phase, patients received long-acting risperidone 50 mg injections every 2 weeks, with 2 weeks of oral risperidone supplementation. Injections of long-acting risperidone 50 mg every 4 weeks followed for up to 48 weeks, without oral supplementation. The primary endpoint was relapse; other assessments included PANSS, CGI-S, adverse event reports, and determination of risperidone and 9-hydroxyrisperidone plasma concentrations.

**Results:**

Twelve patients in the intent-to-treat population (n = 67) met relapse criteria (17.9%). Relapse risk at 1 year was estimated as 22.4%. Non-statistically significant improvements in symptoms (PANSS) and clinical status (CGI-S) at endpoint were observed. The most common adverse events included schizophrenia aggravated not otherwise specified (19.5%), anxiety (16.1%), insomnia (16.1%), and headache (11.5%). There were no unexpected safety and tolerability findings. Mean plasma concentrations for risperidone and 9-hydroxyrisperidone were generally stable during the study.

**Conclusion:**

Once-monthly dosing of long-acting risperidone was well tolerated, associated with a relatively low relapse rate (similar to that reported with other antipsychotics), and maintained the clinically stable baseline status of most patients. Although the results suggest that some symptomatically stable patients with schizophrenia or schizoaffective disorder might be safely managed with long-acting risperidone 50 mg once monthly, these findings alone do not identify which patients will have a sufficient therapeutic benefit nor do they quantify comparative effects of standard and altered dosing. Study limitations (the open-label pilot study design, small sample size, and lack of a concurrent biweekly treatment arm) prevent broad interpretations and extrapolations of results. Controlled studies would be required to support a recommendation for alternative dosing regimens.

## Background

Schizophrenia is a chronic, debilitating disease that is often characterized by frequent relapses associated with exacerbation of psychosis and the need for psychiatric rehospitalization [1]. To prevent relapse, compliance with maintenance antipsychotic medication is critical. Recently, the first long-acting injectable atypical antipsychotic, long-acting risperidone, has become available and is approved for administration every 2 weeks in patients with schizophrenia. In contrast to conventional depots, which are esterified prodrugs delivered in an oil-based solvent, long-acting risperidone is an aqueous-based suspension of microspheres of polylactide coglycolide, a biodegradable carbohydrate copolymer in which risperidone is encapsulated. The release of risperidone from these microspheres starts 3 weeks after the injection, with most of the release occurring during weeks 4 through 6. Steady-state plasma concentrations are reached after 4 injections and are maintained for 4 to 6 weeks after the last injection. Mean steady-state peak concentrations and peak-trough fluctuations are reduced with long-acting risperidone biweekly dosing compared with daily oral risperidone dosing [2], potentially enabling further clinical improvements with long-acting risperidone related to more predictable and stable drug plasma levels [2,3].

Several clinical studies have established the efficacy, safety, and tolerability of long-acting risperidone administered once every 2 weeks in patients with schizophrenia or schizoaffective disorder. Significant improvements in the Positive and Negative Syndrome Scale (PANSS) total score were observed relative to placebo in a 12-week, double-blind, placebo-controlled trial in symptomatic patients, and at endpoint relative to baseline in two 1-year studies in patients with stable symptoms at study entry [4-7]. The effect of long-acting risperidone on relapse has also been studied in a 1-year, double-blind trial comparing long-acting risperidone 25 mg and 50 mg every 2 weeks; relapse and rehospitalization rates were low, at 22% and 10%, respectively, in the 25-mg group; and 15% and 6%, respectively, in the 50-mg group [7]. Long-term treatment has demonstrated good tolerability, with few discontinuations due to adverse events, minimal injection-site reactions, and significant improvements in movement disorder ratings [4,6].

Efficacy and safety results from these studies suggest that dosing of 25 mg up to 50 mg, administered every 2 weeks, provides the optimum risk-to-benefit ratio for long-acting risperidone; however, different approaches for maintenance therapy with long-acting risperidone should be explored. To date, examination of long-acting risperidone using a longer injection interval has not been conducted, although there has been clinical interest in extended-interval dosing regimens. Single-dose pharmacokinetic modeling with long-acting risperidone 50 mg once monthly predicted average plasma concentrations similar to that of 25 mg every 2 weeks, but with higher peak-to-trough fluctuations and lower troughs (Janssen, L.P., data on file), consistent with a previous report [8]. However, because the relationship between medication blood levels and clinical outcomes is not well characterized, a clinical study is needed to assess the utility of such an approach. The objective of this prospective pilot study is to examine the efficacy, safety, tolerability, and pharmacokinetics of maintenance treatment with long-acting risperidone 50 mg once monthly in stable patients with schizophrenia or schizoaffective disorder.

## Materials and methods

This prospective, open-label, 1-year, single-arm, multicenter pilot study was performed between May 2002 and December 2003, and included 12 sites in the United States and Canada. The trial was conducted in accordance with current Good Clinical Practice guidelines and the Declaration of Helsinki and its subsequent revisions. Independent ethics committees or institutional review boards (IRBs) reviewed and approved the final protocol and any amendments to the study.

### Patients

Patients were aged 18 to 65 years with a diagnosis of schizophrenia or schizoaffective disorder according to DSM-IV criteria [9]. Patients were judged to be symptomatically and medically stable, with no other clinically significant medical conditions. All patients received oral risperidone monotherapy at a stable dose (2–6 mg/d) for 8 weeks prior to baseline. Patients were excluded if they had significant symptom exacerbation (requiring hospitalization or acute crisis intervention) in the 8 weeks prior to baseline; were judged by the investigator to be at imminent risk of injury to self, to others, or to property; had a history of substance abuse within the past 6 months or a positive substance test at screening; had impaired hepatic or renal function; were pregnant or breastfeeding; or had received treatment with an oral antipsychotic other than risperidone in the past 8 weeks, depot antipsychotics in the past 6 months, long-acting risperidone in a previous clinical trial, another investigational agent within 30 days, or electroconvulsive therapy within 12 months of baseline. Each patient (or a relative, guardian, or legal representative) provided written informed consent before participating in the study.

### Trial medication

Clinically stable patients receiving oral risperidone for at least 8 weeks (2–6 mg/d) and meeting inclusion criteria were eligible to enter the 52-week treatment phase, which consisted of a 4-week lead-in phase and a 48-week monthly injection and evaluation phase. During the lead-in phase, patients received injections of long-acting risperidone 50 mg every 2 weeks, with 2 weeks of oral risperidone supplementation (2–6 mg/d) after the first long-acting risperidone injection. During the monthly injection and evaluation phase, patients received long-acting risperidone 50 mg every 4 weeks; oral risperidone supplementation was not permitted. Long-acting risperidone was administered by intramuscular gluteal injection. Patients meeting relapse criteria had the option of receiving 75 mg once monthly, with oral risperidone supplementation during the first 3 weeks after the first 75-mg injection.

Patients were allowed to continue any antidepressants, anxiolytics, or mood stabilizers if they had been receiving stable doses for at least 8 weeks before baseline. Other than oral risperidone treatment as described above, no other antipsychotics were allowed. Zolpidem or zaleplon were allowed for insomnia, lorazepam was permitted for agitation or severe restlessness, and benztropine mesylate was permitted for extrapyramidal symptoms.

### Assessments

The primary efficacy parameter was time to relapse in the intent-to-treat (ITT) population. The relapse incidences at each follow-up time were estimated by Kaplan-Meier survival analysis. Relapse criteria were similar to those reported previously [1] and were defined by any one of the following: psychiatric hospitalization due to worsening symptomatology; a significant increase in the level of psychiatric care required (eg, significant crisis intervention needed to avert hospitalization, clinically notable increases in the frequency or intensity of patient contact required to maintain outpatient status), and a 25% increase from baseline in the PANSS total score, occurring within 2 weeks of one another; substantial clinical deterioration, as indicated by a score of 6 ("much worse") or 7 ("very much worse") on the Clinical Global Impressions of Change (CGI-C) scale; or deliberate self-injury, suicidal or homicidal ideation that is clinically significant as determined by the investigator, or violent behavior resulting in clinically significant injury to another person or in property damage. Efficacy parameters included the PANSS total [10] and PANSS factor scores [11] and the Clinical Global Impressions of Severity (CGI-S) scale [12], which were assessed at baseline, weeks 2 and 4, and every 4 weeks thereafter.

Safety was assessed via regular monitoring of treatment-emergent adverse events, laboratory tests, and vital signs. The presence and severity of movement disorders were evaluated by the Extrapyramidal Symptom Rating Scale (ESRS)[13] and the Abnormal Involuntary Movement Scale (AIMS) [12]. Treatment-emergent adverse events and vital signs were monitored at each study visit; laboratory tests were performed at screening, baseline, and endpoint; the ESRS was performed at baseline and weeks 4, 8, 12, 16, 28, 40, and 52 (endpoint); and the AIMS was performed at baseline and endpoint.

All patients were encouraged to return for their long-acting risperidone injection within ± 3 days of their regularly scheduled visit. Partial compliance/noncompliance with injections was defined as receiving more than 25% of injections outside this 3-day window for any of the study time points.

Venous blood samples were taken for drug level measurements; 5-mL trough samples were drawn from all patients immediately before each injection at weeks 0, 2, and 4, and every 4 weeks thereafter, including endpoint. More intensive sampling occurred in a subset of patients (n = 18), from whom blood was drawn every 4 days between weeks 24 and 28. Plasma concentrations of risperidone and its active metabolite, 9-hydroxyrisperidone, were determined by a validated liquid chromatography tandem mass spectrometry (LC-MS/MS) method [14]. The following pharmacokinetic parameters of the sum of risperidone plus 9-hydroxy-risperidone were determined during the period of intensive sampling in the subset of 18 patients: apparent minimum plasma concentration at steady state (C_min,ss_); apparent maximum plasma concentration at steady state (C_max,ss_); time to reach the maximum plasma concentration at steady state (t_max,ss_); area under the plasma concentration-time curve during a 4-week dosing interval (τ) at steady state (AUCτ); average plasma concentration at steady state, calculated as AUCτ divided by the dosing interval (τ) (C_avg,ss_); fluctuation index (Fl), ie, percentage fluctuation, calculated as 100* [(C_max_-C_min_)/C_avg,ss_]; and peak-to-trough ratios (C_max_/C_min_).

### Data analysis

The sample size was not based on any statistical calculations for power, as this was an exploratory study. However, a sample size of 80 subjects entering the study was deemed sufficient to allow for a preliminary exploration of safety, tolerability, pharmacokinetics, and efficacy in the context of an open-label, single-arm investigation, with consideration of stratification factors described below. Efficacy analyses were performed on the ITT population, prospectively defined as patients who received at least 1 injection of long-acting risperidone and who had at least 1 efficacy evaluation during the monthly injection and evaluation period of treatment (after week 4 of the study). Safety evaluations were performed for all patients who received at least 1 injection of long-acting risperidone. There were no adjustments made for multiplicity.

Time to relapse was determined using Kaplan-Meier methodology. Ninety-five percent confidence intervals of the Kaplan-Meier estimates of the probability of relapse were obtained using Greenwood's formula [15] for the standard errors at 3 months, 6 months, and 1 year. For the analysis of relapse, subjects were prospectively stratified on the basis of the time since last hospitalization into 2 groups: between 2 and 6 months (56–167 days, inclusive); and more than 6 months (168 days or longer), from the first dose of long-acting risperidone. A post hoc analysis assessed relapse in patients with at least 1 efficacy evaluation after week 8 in order to examine the effect of the once-monthly administration only.

For other efficacy variables, changes from baseline and observed values were summarized descriptively, including 95% confidence intervals at each time of evaluation and at endpoint. A paired *t *test for the difference between baseline and endpoint was used for secondary parameters.

The pharmacokinetic analysis of risperidone plus 9-hydroxyrisperidone was based on data from 771 of 957 samples collected from 87 patients. Excluded samples included those taken outside the ranges for inclusion and unscheduled samples (n = 75), samples taken while the subject was taking oral risperidone cotherapy (n = 45), and samples taken after injection with 75-mg long-acting risperidone (n = 66). Descriptive statistics were calculated at each scheduled sampling time and for steady-state pharmacokinetic parameters during the intensive sampling time between week 24 and week 28. Plasma concentrations below the limit of quantification (ie, <0.1 ng/mL for risperidone and 9-hydroxyrisperidone for most samples, <0.2 ng/mL for 2 samples) were set to zero. Plots of mean (± SD) plasma concentrations versus scheduled time points are presented for the sum of risperidone plus 9-hydroxyrisperidone.

## Results

**Figure 1:**
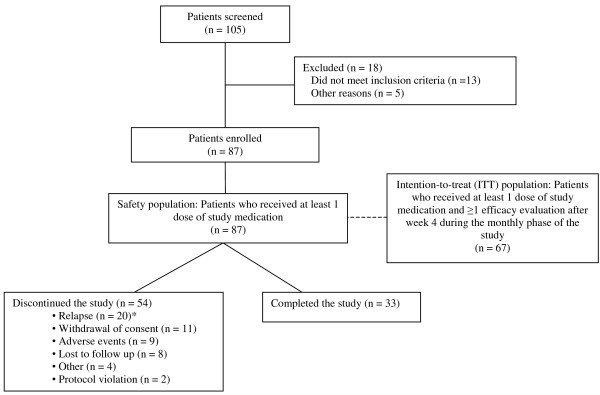
Study disposition. *Eight patients in the safety population were not part of the prospectively defined ITT population (n = 67) for which the primary endpoint, relapse incidence, was calculated. Twenty patients in the safety population, versus 12 patients in the ITT population, relapsed.

A total of 87 patients participated in this pilot study and were included in the safety analyses. The mean (± SD) risperidone oral dose was 3.9 (1.2) mg/d during the 2-week screening period and 4.0 (1.5) mg/d during the long-acting risperidone 4-week lead-in period. The mean patient age (± SD) was 39.8 ± 10.3 years, 58 (66.7%) were male, and the majority (78.2%) had a diagnosis of schizophrenia. Thirty-five (40.2%) were Caucasian, 31 (35.6%) were Black, 19 (21.8%) were Hispanic, and 2 (2.3%) were Other. Reasons for study discontinuation, including relapse, are listed in Figure 1. Overall, 75.9% of the patients were compliant, as defined in the Methods section.

### Efficacy

**Figure 2:**
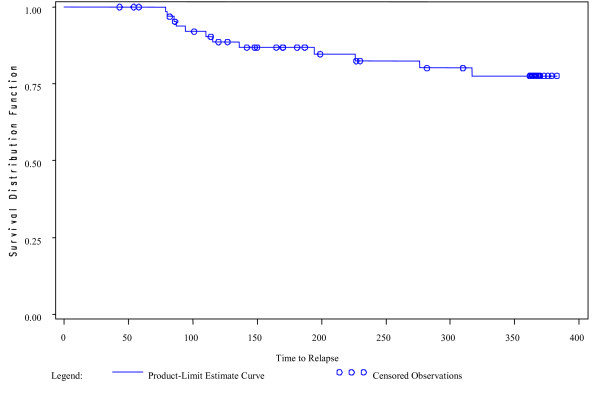
Kaplan-Meier survival curve for relapse, intention-to-treat (ITT) population.

At the end of the study, 12 patients (17.9%) in the ITT population (n = 67) had relapsed. Six patients required psychiatric hospitalization and 6 patients experienced substantial clinical deterioration. The Kaplan-Meier estimate of the risk of relapse in the ITT population at 1 year was 22.4% (95% confidence interval, 10.9%–33.9%) (Figure 2). Of the 22 patients in the ITT population with a recent hospitalization prior to study entry, 4 (18.2%) met criteria for relapse during the trial. Of the 45 patients with a hospitalization more than 167 days from the first dose of long-acting risperidone, 8 (17.8%) met criteria for relapse. The time to relapse curves were similar for these 2 groups. Median time to relapse could not be calculated since fewer than 50% of subjects relapsed over the 52 weeks of the trial.

A post hoc analysis was performed to assess relapse in patients with at least 1 efficacy evaluation after week 8 to examine the effect of once-monthly dosing only. The number who relapsed (n = 12) and the Kaplan-Meier estimate of the risk of relapse were the same as in the ITT population because, even though there were 3 fewer patients in this post hoc population (n = 64), none of those 3 patients met relapse criteria (1 withdrew consent and 2 discontinued owing to adverse events).

The mean PANSS total score improved over the course of the study in this population of symptomatically stable patients; the improvement was significant at all time points, including week 52 (completers), but not at endpoint (LOCF analysis; Table 1). Analysis of PANSS factor scores indicated that all five (positive, negative, anxiety/depression, uncontrolled hostility/excitement, and disorganized thought factor scores) were significantly improved (*P *< .01) at week 52, but not at endpoint (Table 1).

**Table 1:** Positive and Negative Syndrome Scale (PANSS) Scores at Baseline, Week 52, and Endpoint (Intention-to-Treat Population)

**PANSS, mean ± SD**	**Baseline (N = 67)**	**Week 52 (n = 31)**	**LOCF Endpoint (N = 67)**
Total	65.4 ± 13.5	57.5 ± 12.7	61.7 ± 17.9
		*P *< .0001	*P *= .087
Positive symptoms	18.1 ± 5.3	15.6 ± 4.5	17.3 ± 6.2
		*P *< .0001	*P *= .233
Negative symptoms	17.4 ± 5.4	16.0 ± 4.4	16.1 ± 5.4
		*P *= .007	*P *= .071
Anxiety/depression	9.1 ± 3.0	7.3 ± 2.7	8.5 ± 3.7
		*P *= .002	*P *= .133
Disorganized thought	14.4 ± 4.1	13.4 ± 3.8	13.9 ± 4.2
		*P *= .001	*P *= .337
Uncontrolled hostility/excitement	6.3 ± 2.4	5.3 ± 1.7	5.9 ± 2.4
		*P *= .001	*P *= .278

*P *values represent significance of change from baseline, paired *t *test. LOCF indicates last observation carried forward.

Mean CGI-S scores improved over the course of the study. The improvement was significant at week 52 (completers) compared with baseline (*P *= .0002), but not at endpoint (LOCF analysis). The number (%) of patients with a categorical CGI-S rating of not ill to mildly ill increased from 34 (50.8%) at baseline to 42 (62.7%) at endpoint (Table 2).

**Table 2:** Clinical Global Impressions of Severity (CGI-S) Categorical Ratings at Baseline and Endpoint (Intention-to-Treat Population)

**CGI-S Rating**	**Baseline (N = 67) n (%)**	**Endpoint (n = 67) n (%)**
Normal	0	0
Borderline mentally ill	2 (3.0)	11 (16.4)
Mildly ill	32 (47.8)	31 (46.3)
Moderately ill	25 (37.3)	13 (19.4)
Markedly ill	8 (11.9)	11 (16.4)
Severely ill	0	1 (1.5)

Eight subjects in the ITT population relapsed while receiving 50 mg once monthly, and then continued in the study with the 75 mg once monthly dose. Efficacy data are available for 6 of these subjects. Mean total PANSS scores improved from 88.4 ± 21.8 at baseline (baseline was defined at the start of the 75 mg dosing period) to 65.8 ± 10.7 at endpoint. The mean CGI-S score also improved, from 5.0 ± 0.6 at baseline to 4.2 ± 1.2 at endpoint. Although positive, these results should be interpreted with caution due to the small number of subjects for whom data were available.

### Safety and tolerability

A total of 67 patients (77.0%) experienced at least 1 adverse event during the study, and 9 patients (10.3%) discontinued the study owing to adverse events. The majority of adverse events reported were mild or moderate in severity. The most common adverse events (reported by more than 10% of subjects) included schizophrenia aggravated not otherwise specified (NOS; 19.5%), anxiety (16.1%), insomnia (16.1%), and headache (11.5%). The incidence of movement disorder adverse events was low: tremor (4.6%), dyskinesia (3.4%), akathisia (2.3%), hypertonia (2.3%), and dystonia (1.1%). Only 2 patients (2.3%) reported pain at the injection site.

There were 25 reports of serious adverse events (SAEs), representing 18 patients, during the course of the trial. No deaths occurred in this study. Of the 13 SAEs rated as severe, 1 (schizophrenia NOS aggravated) was categorized as possibly and 1 (weight decreased) as very likely related to the trial medication.

ESRS subscale scores of particular interest include the overall movement disorder subjective score, the physician's examination of parkinsonism, and the physician's examination of dyskinesia. Mean scores were unchanged or improved at endpoint in each of these subscales, with mean (± SD) scores (baseline, endpoint) as follows: movement disorder subjective score: 1.7 (2.3), 1.3 (2.1); physician's examination of parkinsonism: 2.3 (4.7), 0.9 (2.0); and physician's examination of dyskinesia: 1.0 (2.1), 0.9 (2.2). Improvement in the parkinsonism subscale was significant at week 52 (*P *< .05) and endpoint (*P *< .01). Mean scores on the dystonia and akathisia subscales were low (<1) both at baseline and endpoint. The mean (± SD) total AIMS score at baseline was low, 1.5 (2.8), and improved during the study, with a mean (± SD) change from baseline at week 52 of -0.9 (2.9) and at endpoint of -0.2 (3.7); the change from baseline was not significant at either time point (*P *= .090 at week 52; *P *= .662 at endpoint).

There were no clinically meaningful changes on mean laboratory values, mean vital signs, or electrocardiogram (ECG) parameters. The mean (± SD) random blood glucose level was 109.8 (35.6) mg/dL at baseline and remained stable throughout the study, with a mean (± SD) change of 5.0 (34.5) mg/dL from baseline at week 52. The mean (± SD) prolactin level at baseline was 43.1 (41.0) ng/mL and remained stable during the study; there was a mean decrease of 1.1 (37.64) ng/mL from baseline at week 52. The mean (± SD) body weight at baseline was 92.8 (24.5) kg, with a mean change of -0.3 (5.5) kg from baseline to endpoint.

### Pharmacokinetic analysis

**Figure 3:**
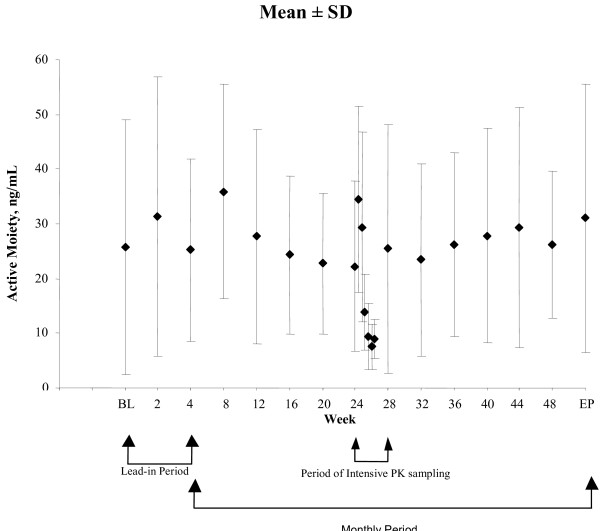
Mean (± SD) plasma concentrations of risperidone plus 9-hydroxyrisperidone by study week. Pharmacokinetic (PK) analyses are based on 771 samples from 87 patients. Intensive pharmacokinetic data are based on 7 samples planned between weeks 24 and 28 (1 every 4 days) from 18 patients.

The pharmacokinetic analysis of risperidone plus 9-hydroxyrisperidone was based on data from 771 samples from 87 patients. Mean plasma concentrations for risperidone plus 9-hydroxyrisperidone were generally stable throughout the study (Figure 3). During steady state, obtained with samples from 18 patients, the average plasma concentration of risperidone plus 9-hydroxyrisperidone was 17.5 (6.1) ng/mL, and the fluctuation index was 199% (55.8) (Table 3).

**Table 3:** Steady-State Pharmacokinetic Parameters of Risperidone Plus 9-hydroxyrisperidone with 50 mg Once-Monthly or 25 mg Biweekly

	50 mg Once Monthly*		**25 mg Biweekly [2]**	
**Parameter**	**n**	**Mean ± SD**	**n**	**Mean ± SD**

C_min,ss_, ng/mL	18	6.21 ± 3.1	21	11.3 ± 4.5
C_max,ss_, ng/mL	18	40.4 ± 15.3	21	22.7 ± 9.2
t_max,ss_, h	18	240.2 ± 217.2	21	NA
AUCτ, ng·h/mL	17	12,027 ± 4241	21	5303
C_avg,ss_, ng/mL	17	17.5 ± 6.1	21	15.8
FI, %	17	199 ± 55.8	21	69 ± 44
C_max_/C_min _ratio	18	7.4 ± 2.8	21	2.4 ± 1.8

NA indicates not available.

*Samples acquired between weeks 24 and 28.

AUCτ: the dosing interval (τ) is 4 weeks for the present study and 2 weeks for Eerdekens M, et al.

## Discussion

Short- and long-term studies of long-acting risperidone have suggested an optimal population benefit/risk ratio at doses of 25 mg up to 50 mg administered every 2 weeks, the dosing interval for which this product has been approved [4-7]; however, there is clinical interest in the possibility of a longer injection schedule, and models derived from single-dose pharmacokinetic data at the 50-mg dose suggested that 50 mg every 4 weeks would result in average plasma concentrations similar to those with 25 mg given every 2 weeks, albeit with higher peak-to-trough fluctuations and lower trough levels (Janssen Pharmaceutica, Inc., data on file). To date, there are no published reports supporting alternative dosing intervals with long-acting injectable risperidone, and important clinical questions on the associated tolerability, safety, and efficacy associated with a longer injection interval remain unanswered. We explored these issues as well as the pharmacokinetics of once-monthly administration of long-acting risperidone 50 mg in a small pilot study of stable patients with schizophrenia or schizoaffective disorder. Since this is the first clinical study assessing a monthly dosing regimen, a pilot approach was chosen to minimize potential risk to a larger number of patients.

The relapse rate was relatively low (17.9%) in this 1-year study of patients clinically stable at study entry receiving once-monthly long-acting risperidone 50 mg; the Kaplan-Meier estimate of the risk of relapse at 1 year was 22.4%. No meaningful difference was observed in the rate of relapse between subjects with a recent hospitalization and those with a longer time since last hospitalization. While it is difficult to make comparisons across studies for many reasons, including differences in relapse criteria, this relapse incidence is similar to those found in US-based clinical trials examining long-acting injectable antipsychotics [16-20]. A head-to-head comparison of risperidone and haloperidol found 1-year relapse rates were 25.4% for patients receiving risperidone 2–8 mg/d vs 39.9% for haloperidol 5–20 mg/d [1]. One-year rates of relapse for ziprasidone were 43%, 35% and 36% for doses of 40, 80, and 160 mg/d, respectively [20], whereas treatment with fluphenazine decanoate 12.5–100 mg/3 weeks was associated with a 1-year relapse rate of 28% [19]. Other relapse data for long-acting risperidone are available from a randomized, double-blind, 52-week study of 2 doses of long-acting risperidone (25 or 50 mg) administered every 2 weeks. One-year relapse incidence was 21.6% and 14.9% for patients receiving the 25 mg and 50 mg doses, respectively [7].

Although patients were symptomatically stable at baseline, additional efficacy analyses showed that significant improvements were noted on the PANSS and the CGI-S scores throughout the study, except at endpoint, demonstrating maintenance or improvement of symptom control. In this study, the mean change from baseline in the PANSS total score at endpoint was -3.7 (17.7); *P *= .087. Significant improvements in PANSS total scores were observed at endpoint in 2 other long-term studies of long-acting risperidone [4,7]. In a 1-year, open-label study that included 561 patients, the mean (± SE) change in PANSS total score at endpoint was -6.1 (0.7), *P *< 0.01 vs baseline [4]. In a randomized, double-blind, 52-week study that included 323 patients, the mean (± SD) change in PANSS total score was -4.9 (16.8), *P *≤ .001 vs baseline [7]. Baseline total PANSS scores in this study and the other 1-year studies [4,7] were similar. The smaller numbers of patients in the current study may explain the differences in significance in PANSS total scores at endpoint. As relapse prevention and symptom control are key goals in the treatment of chronic schizophrenia, these results suggest that some symptomatically stable patients with schizophrenia or schizoaffective disorder might be safely managed with long-acting risperidone 50 mg once monthly; however, these data do not allow us to distinguish those patients for whom this dosing regimen may be sufficient versus those for whom it may be suboptimal, nor does this pilot design permit firm conclusions concerning comparative efficacy or tolerability of the alternative dosing regimen.

In addition to a relatively low relapse rate and maintenance of effect in most patients, other results of this pilot study suggested that long-acting risperidone 50 mg once monthly was well tolerated, with a safety profile similar to that reported for long-acting risperidone administered on a biweekly schedule [4,6,7]. Movement disorder ratings were all low at baseline in this population and were unchanged or improved during the study. There were no clinically meaningful changes in mean laboratory values, vital signs, or ECG parameters. The mean body weight decreased modestly over the course of this study. In contrast, small mean body weight increases were noted in two other 1-year studies of long-acting risperidone in which risperidone was administered biweekly [4,7]. Overall, this pilot data suggests that the monthly dosing regimen may not have greater liability regarding safety or tolerability compared with biweekly dosing.

Single-dose pharmacokinetic modeling with long-acting risperidone 50 mg once monthly predicted average plasma concentrations similar to those seen with 25 mg given every 2 weeks, but with higher peak-to-trough fluctuations and lower troughs, resulting in intermittent periods of low exposure (Janssen, L.P., data on file). Consistent with those data, the pharmacokinetic results showed more variability with once-monthly compared with biweekly dosing. Indeed, C_min _values associated with the 50-mg monthly injections were approximately half of the steady-state values measured in another study of long-acting risperidone injection 25 mg administered biweekly [2]. Further, the percentage fluctuation and the C_max_-to-C_min _ratios were approximately 3 times higher after monthly injections than after biweekly injections;[2] however, the average exposure to risperidone plus 9-hydroxyrisperidone was comparable between once-monthly dosing and historical data from biweekly dosing. While previous reports have suggested that greater plasma level fluctuations may be associated with poorer tolerability [2,3], this was not supported in the overall results from this pilot study.

## Conclusion

The intent of this pilot study was to begin addressing a critical clinical question regarding alternative dosing frequency with long-acting risperidone, particularly once-monthly administration. Once-monthly dosing of long-acting risperidone appeared to maintain the baseline symptomatic status of stable patients with schizophrenia or schizoaffective disorder, with no unexpected safety and tolerability findings. While the results are encouraging, interpretations are limited by several factors, including the open-label pilot study design, small sample size, and lack of a concurrent biweekly treatment arm. Further, additional information on illness course in patients prior to risperidone therapy would also have been helpful for interpreting the study findings.

Despite the limitations of the trial, the findings support a possible role for risperidone long-acting 50 mg once monthly in certain stable patients with schizophrenia or schizoaffective disorder. A controlled trial evaluating both a biweekly and monthly administration regimen would be necessary, however, to further explore the clinical impact of once-monthly relative to biweekly dosing. Results from this trial will help provide direction for future controlled studies to validate alternative dosing regimens with long-acting risperidone.

## Competing interests

Funding for this study was provided by Janssen, L.P.
